# Comprehensive Analysis in the Nutritional Composition, Bioactive Contents, and Antioxidant Capacity of Walnut (*Juglans regia* L.) Male Flowers

**DOI:** 10.3390/foods14244250

**Published:** 2025-12-10

**Authors:** Fengmei Lei, Yuqing Liu, Tianmeng Shi, Lufeng Zhang, Yanqing Zhang, Lianjun Song, Xianqing Huang, Ning Li, Mingjing Li, Yue Shen, Qian Li

**Affiliations:** 1 Henan Engineering Technology Research Center of Food Processing and Circulation Safety Control, College of Food Science and Technology, Henan Agricultural University, Zhengzhou 450002, China; lfm0522@126.com (F.L.); tgcf111@126.com (T.S.); zhangyanqing0224@163.com (Y.Z.); slj69@126.com (L.S.); hxq8210@126.com (X.H.); lining8028@126.com (N.L.); 2 Key Laboratory of Natural Products, Henan Academy of Sciences, Zhengzhou 450002, China; myjoe00@163.com; 3 Yuanrong Agriculture Co., Ltd., Sanmenxia 472200, China; 18236736781@163.com (L.Z.); bwjj88@126.com (M.L.)

**Keywords:** *Juglans regia* L., flower, amino acid, mineral composition, antioxidant capacity

## Abstract

Walnut male flowers (WMFs) are important by-products of walnut production. Studies on the nutritional quality of WMFs have predominantly focused on a single variety or region, and scientific information on different varieties is limited. In this study, ten walnut male flower (WMF) samples were evaluated and compared to assess their nutritional composition, bioactive contents, and antioxidant capacity. All WMF varieties were rich in protein, minerals, and amino acids, with leucine being the most abundant amino acid. All varieties exhibited low fat content and a favorable Na/K ratio. Additionally, they contained high levels of polyphenols and flavonoids, which were associated with strong antioxidant capacity. The variety seemed to exert a greater influence than the region on the nutritional composition and bioactive contents of WMFs. Moreover, polysaccharide, starch, soluble sugar, polyphenol, and fat might serve as potential markers for distinguishing different WMF varieties. This study provides a reference for the development and utilization of WMFs.

## 1. Introduction

Walnuts (*Juglans regia* L.) have a long history of cultivation worldwide. Walnut flowers, specifically the walnut male flowers, are significant by-products of walnut production. China is one of the major walnut-cultivating countries, contributing approximately 50% of global production [1]. With the rapid development of the walnut industry, the planting area has increased steadily. In 2021, the walnut planting area in China reached 7.4549 million hectares, yielding a total output of 5.4035 million tons [2]. WMFs influence both walnut growth and fruit quality, and a significant quantity is discarded during production. Although small amounts of WMFs are consumed as food in some regions, the majority are treated as waste [3]. These by-products possess substantial commercial value and potential for walnut industrial development.

In recent years, WMFs have garnered considerable attention due to their nutritional value and biological properties. However, most research has primarily focused on individual varieties, with relatively little attention given to the examination of multiple varieties. For example, Wang et al. [4] showed that the Qianhe-7 WMFs were rich in protein, carbohydrate, and minerals. The methanol extract of WMFs from the Doon Fidin variety, sourced from the Indian Himalayan region, was rich in polyphenols (129.76 mg GAE/g DW) and flavonoids (144.62 mg RE/g DW), exhibiting excellent antioxidant and bacteriostatic properties [5,6]. Jin et al. [7] demonstrated that WMFs (from Dayao City, Yunnan Province), possess the capacity to improve the intestinal microbial environment. Additionally, some studies have indicated that WMFs can serve as enzyme inhibitors to reduce enzyme activity and possess strong biological activity by lowering blood glucose levels [8]. In conclusion, it is evident that there is considerable potential for further studies on WMFs.

Previous studies have indicated that both variety and region significantly affect the nutritional composition and antioxidant activity of quinoa [9,10]. A similar comprehensive approach, combining proximate composition, mineral analysis, and antioxidant profiling, has been successfully employed to screen and evaluate different plant varieties, such as quinoa [9]. While previous studies [4,5,6,7,8] on WMFs have typically focused on single varieties, the influence of genetic and geographical factors across multiple varieties remains limited. This study comprehensively evaluated ten WMF samples from two major walnut-producing regions in China, Henan and Xinjiang, including the unique same variety Tuhetao from both regions to directly analyze the effects of variety and region. We aimed not only to evaluate their nutritional composition and bioactive prifiles (flavonoid, polyphenol and antioxidant properties), but also to determine the dominant factor govering these traits. Thus, multivariate statistics, including principal component analysis (PCA) and partial least squares-discriminant analysis (PLS-DA) were used to identify potential discriminative markers and elucidate the complex relationships between nutritional composition, bioactive content, and in vitro antioxidant capacity. This study aims to provide a basis for the targeted development and utilization of WMFs.

## 2. Materials and Methods

### 2.1. Raw Materials

The detailed information of the ten WMF samples is provided: LL, XL, THT-HN, YF, and LH were collected in Sanmenxia City, Henan Province, while XF, SS, THT-XJ, 185, and X2 were obtained from Aksu City, Xinjiang Uyghur Autonomous Region. Notably, the two THT samples (THT-HN and THT-XJ) were collected from Henan and Xinjiang, respectively. All samples were naturally air-dried in a well-ventilated, shaded environment (at approximately 20–25 °C and 40–60% relative humidity). After being naturally air-dried, the samples were ground and passed through a 60-mesh sieve. The resulting powder (≤60 mesh) was collected and stored at 4 °C for subsequent analysis.

### 2.2. Chemical Reagents

Gallic acid, rutin, glucose standard, alkaline protease, α-amylase, 2,2-diphenyl-1-picrylhydrazyl (DPPH), and 2,2′-Azinobis-(3-ethyl-benzothiazoline-6-sulfonic acid) (ABTS) were obtained from McLean Biochemical Technology Co., Ltd. (Shanghai, China). The FRAP assay kit was purchased from Solepol Technology Co., Ltd. (Beijing, China). Hydrochloric acid, sulfuric acid, petroleum ether (boiling range 30–60 °C), Folin–Ciocalteu reagent, and phenol were procured from Aladdin Biochemical Technology Co., Ltd. (Shanghai, China). Boric acid, sodium nitrite, sodium carbonate, aluminum chloride, sodium hydroxide, copper sulfate, and potassium sulfate were acquired from Sinopharm Chemical Reagent Co., Ltd. (Beijing, China). Anhydrous ethanol was obtained from Xilong Scientific Co., Ltd. (Shanghai, China).

### 2.3. Determination of Protein, Moisture, and Ash

Total nitrogen content was determined using the method of Kan et al. [11], with a nitrogen-to-protein conversion factor of 6.25. The moisture content of the naturally air-dried samples was determined by drying them at 105 ± 1 °C to constant weight. Ash content determination was modified as follows: Pretreated WMF samples were partially carbonized on an electric hot plate at low heat until smokeless, then transferred to a muffle furnace and ashed at 550 °C for 4 h. After cooling to approximately 200 °C, the samples were removed, placed in a desiccator and cooled for 30 min, weighed, and re-ashed until a constant mass was achieved (a difference of <2 mg between consecutive weighings).

### 2.4. Determination of Fat and Total Dietary Fiber

Crude fat was analyzed gravimetrically via petroleum ether extraction using a Soxhlet apparatus, and total dietary fiber was determined by the enzymatic-gravimetric method [9].

### 2.5. Determination of Digestible Carbohydrates

Digestible carbohydrate content was calculated according to the method of de Medeiros et al. [12]. Digestible carbohydrate content (g/100g) = 100 − (crude protein (g/100g) + crude fat (g/100g) + ash (g/100g) + dietary fiber (g/100g)).

### 2.6. Determination of Amino Acids and Calculation of Amino Acid Scores (AAS)

Adapted from Labba et al. [13]. 200 mg of the WMF sample was hydrolyzed with HCl (6 M, 30 mL) at 110 °C for 24 h. After filtration, the hydrolysate was diluted with HCl (6 M, 50 mL). Subsequently, 3 mL of the solution was evaporated to dryness, redissolved in HCl (0.02 M, 30 mL), filtered through a 0.22 μm membrane, and analyzed using an amino acid analyzer.

AAS were calculated according to the FAO/WHO 2013 standard [14] as follows: AAS = (amount of an amino acid in the sample protein in mg/g protein)/(amount of the same amino acid in the reference pattern in mg/g protein).

### 2.7. Measurement of Mineral Contents

Mineral element analysis was performed using inductively coupled plasma mass spectrometry (ICP-MS, Agilent 7900, Santa Clara, CA, USA) according to the method of Dai et al. [15]. Briefly, the WMF sample was digested with ultrapure HNO_3_ under high-temperature/pressure conditions, cooled, transferred to a 50 mL volumetric flask, and filtered through a 0.45 μm PTFE membrane; this was followed by instrumental analysis.

### 2.8. Determination of Soluble Sugar

The glucose content was determined using the sulfuric acid-phenol method. A standard curve was established with the concentration of the glucose (mg/mL) as the *x*-axis, and the OD value as the *y*-axis (y = 6.0929x + 0.0564; R^2^ = 0.99).

The soluble sugar content was determined using a method modified from Zeng et al. [16]. Briefly, 0.25 g of the WMF sample was mixed with 10 mL distilled water in a centrifuge tube. The mixture was sonicated (20 °C, 200 W, 50 min), centrifuged (8000 rpm, 10 min), and the supernatant was collected. This extraction was repeated twice. The combined supernatants were transferred to a 100 mL volumetric flask and diluted to the mark. The absorbance was measured at 490 nm.

### 2.9. Determination of Polysaccharides

The polysaccharides determination was modified from Zhao et al. [17]. Briefly, polysaccharides were extracted via water extraction-alcohol precipitation: 1 g of the WMF sample was mixed with 50 mL distilled water, extracted at 90 °C for 2 h, and filtered. The supernatant was combined with a fourfold volume 95% (*v*/*v*) ethanol aqueous solution and stored at 4 °C for 24 h to precipitate polysaccharides. The mixture was centrifuged (8000 rpm, 10 min), and the pellet was collected, washed sequentially with 95% (*v*/*v*) ethanol aqueous solution and acetone, and dried. The dried precipitate was redissolved in 5 mL distilled water, sonicated (20 °C, 200 W, 30 min, three cycles), and the supernatant was transferred to a 25 mL volumetric flask and brought to volume. The absorbance was measured at 490 nm using a spectrophotometer.

### 2.10. Determination of Starch

Starch determination was modified from Liberal et al. [18]. Briefly, 0.1 g of the WMF sample was mixed with 5 mL 95% (*v*/*v*) ethanol aqueous solution in a centrifuge tube. The mixture was sonicated (20 °C, 200 W, 15 min), centrifuged (8000 rpm, 10 min), and the supernatant was discarded. The tubes were air-dried in a fume hood to remove residual ethanol. The residue was reconstituted with 10 mL distilled water, heated in a boiling water bath (15 min for gelatinization), cooled to 60 °C, and treated with amylase. Digestion proceeded in a shaker (60 °C, 120 rpm, 1 h) until iodine testing confirmed the absence of starch. The hydrolysate was cooled, filtered, and mixed with 10 mL HCl (2 M). After refluxing in a boiling water bath for 1 h, the solution was neutralized with 20% (*w*/*v*) sodium hydroxide solution after the addition of 2 drops of methyl red indicator. Finally, the mixture was transferred to a 100 mL volumetric flask, brought to volume, and analyzed spectrophotometrically at 490 nm.

### 2.11. Extraction of Phenolic Compounds

The extraction of phenolic compounds was performed according to da Cruz et al. [19]. Briefly, 1 g of the WMF sample was mixed with 25 mL 70% (*v*/*v*) ethanol aqueous solution and sonicated (45 °C, 45 min). The mixture was centrifuged (8000 rpm, 5 min), and the supernatant was collected. This extraction procedure was repeated twice, and the combined supernatants were adjusted to 50 mL in a volumetric flask. All extractions were performed in triplicate.

### 2.12. Determination of Total Phenolic Content (TPC) and Total Flavonoid Content (TFC)

TPC and TFC in WMFs were determined according to da Cruz et al. using UV spectrophotometry [19]. The linear regression equation for polyphenols based on the gallic acid standard curves had an R^2^ = 0.99 with equation y = 9.0607x + 0.0100, and the final results were expressed as milligrams of gallic acid equivalents (GAE) per gram of dry weight (mg GAE/g DW). The Folin–Ciocalteu method was employed with gallic acid as the standard. Briefly, 1 mL of the WMF extract was transferred to a 10 mL volumetric flask, mixed with 0.3 mL Folin–Ciocalteu reagent (0.2 M), vortexed, and incubated for 5 min. Subsequently, 3 mL of 7.5% (*w*/*v*) sodium carbonate solution was added, vortexed again, and incubated for 5 min. The solution was diluted to volume with distilled water, mixed thoroughly, and incubated in a 50 °C water bath for 10 min. The absorbance was measured at 765 nm.

The TFC was determined using the regression equation of the calibration curve (y = 10.8770x + 0.0114; R^2^ = 0.99), reported in rutin equivalents (RE) as milligrams per gram of dry weight (mg RE/g DW). Specifically, 2 mL of WMF extract was mixed with 1 mL 5% (*w*/*v*) sodium nitrite solution. After vortexing and incubation (6 min), 1 mL of 10% (*w*/*v*) aluminum nitrate solution was added, followed by another 6 min incubation. Subsequently, 10 mL of 4% (*w*/*v*) sodium hydroxide solution was added. The solution was diluted to volume with distilled water, vortexed, and incubated for 10 min. The absorbance was measured at 510 nm.

### 2.13. Determination of Antioxidant Capacity

#### 2.13.1. Determination of DPPH and ABTS

DPPH and ABTS radical scavenging capacity were determined according to Benali et al. [20]. The 25 µL tested samples or standard solution were fully mixed with 175 µL DPPH solution in a 96-well plate. The absorbance value was measured at 517 nm after a 30 min reaction in the dark at 25 °C. The ABTS^+^ stock solution was prepared at a concentration of 7 mM using potassium persulfate (2.45 mM). The solution was kept in the dark at room temperature for 12 h. Then, 75 µL of each test sample at various concentrations was mixed with 925 µL of the diluted ABTS^+^ solution. The absorbance was measured at 734 nm. The results expressed as IC_50_ (mg/mL), which is defined as the concentration of the tested material required to inhibit 50% of the initial DPPH and ABTS radical concentration.

#### 2.13.2. Determination of Ferric Reducing Antioxidant Power (FRAP)

The FRAP assay was performed according to Chen et al. [21]. The FRAP reagent was prepared by mixing TPTZ (2,4,6-tripyridyl-s-triazine) solution (10 mM), acetate buffer (3.1 g C_2_H_3_NaO_2_·3H_2_O and 16 m C_2_H_4_O_2_), and FeCl_3_·6H_2_O solution (20 mM)at a volume ratio of 1:10:1. Then, 180 μL of the FRAP working solution, 5 μL of distilled water, and samples of different concentrations (0.05, 0.1, 0.2, 0.4, 0.6, 0.8 mmol/L) or FeSO_4_ standard solution were gently mixed in each detection hole of a 96-well plate. After incubation at 37 °C for 5 min, the absorbance was measured at 593 nm. All measurements were determined in triplicate. The linear regression equation was based on the standard curve prepared with FeSO_4_ (y = 1.5360x + 0.0243; R^2^ = 0.99), and the result was expressed as FeSO_4_ standard-equivalent concentration (mmol/L).

### 2.14. Statistical Analysis

Experimental data were processed using Microsoft Excel. Statistical analyses were performed with SPSS 19.0 (IBM Corp., Armonk, NY, USA). Pearson correlation coefficients between bioactive compounds and antioxidant capacity were calculated, with statistical significance set at *p* < 0.05. PCA and PLS-DA were performed on nutritional composition and bioactive contents using SIMCA 14.1 (Umetrics, Umeå, Sweden) for comprehensive statistical evaluations. Data visualization was conducted using Origin 2021 (OriginLab, Northampton, MA, USA) and SIMCA 14.1 (Umetrics, Umeå, Sweden). All data were expressed on a dry weight (DW) basis, except for moisture content, which was expressed on a fresh weight (FW) basis.

## 3. Results and Discussion

### 3.1. Protein

**Figure 1:**
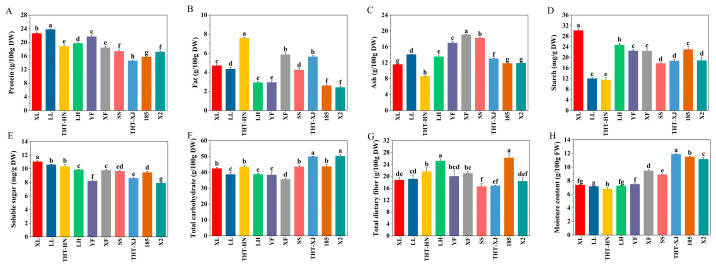
(**A**) Protein, (**B**) fat, (**C**) ash, (**D**) starch, (**E**) soluble sugar, (**F**) total carbohydrate, (**G**) total dietary fiber, and (**H**) moisture content of different samples of WMFs. Note: All data are expressed as the mean ± standard deviation of triplicate measurements. Different superscript letters are significantly different *p* < 0.05.

Proteins are essential components in human biological processes. The protein content of WMFs ranged from 14.57 to 23.79 g/100 g DW (Figure 1A), which was significantly higher than the values reported for Japanese buckwheat (13.27 g/100 g DW) and the flower of *Sophora japonica* L. (11.65 g/100 g DW) [22,23]. Among the WMF samples, LL showed the highest protein content at 23.79 g/100 g DW, followed by XL at 22.62 g/100 g DW, while THT-XJ exhibited the lowest level. Notably, LL contained approximately 1.6-fold higher protein content than THT-XJ, indicating significant varietal differences in protein levels. Previous studies have reported protein content ranging from 26.2 to 32.8 g/100 g DW in faba bean varieties and from 11 to 19 g/100 g DW in quinoa seeds [13,24], indicating that WMFs serve as a competitive protein source among plant materials.

Varieties from Henan exhibited protein contents ranging from 18.87 to 23.79 g/100 g DW, which were significantly higher than the 16 g/100 g DW reported for Indian wheat [25]. Notably, the protein levels in LL and XL (22.62–23.79 g/100 g DW) were comparable to those of meat proteins (22 g/100 g DW) [26]. In contrast, varieties from Xinjiang demonstrated lower protein content, ranging from 14.57 to 18.41 g/100 g DW. This variation may be attributed to geographical, soil, climatic, and biological factors [9].

To determine whether the region or variety exerts a greater influence on WMF protein content, samples of the same WMF variety cultivated in Xinjiang and Henan were analyzed. The results revealed statistically significant differences (*p* < 0.05) in protein content between geographically distinct cultivation areas. Specifically, THT-HN contained 18.87 g/100 g DW protein, whereas THT-XJ contained 14.57 g/100 g DW. These differences may be attributed to geographical and environmental factors [10].

### 3.2. Fat

The fat content of WMFs ranged from 2.40 to 7.58 g/100 g DW (Figure 1B). Among these, THT-HN exhibited the highest fat content at 7.58 g/100 g DW, while X2 showed the lowest at 2.40 g/100 g DW. Compared to the fat content of soybean (14.92–22.19 g/100 g DW) and black soybean (14.13–20.45 g/100 g DW) [11], WMFs had a lower fat content. The fat content of the Henan varieties ranged from 2.91 to 7.58 g/100 g DW, whereas Xinjiang varieties ranged from 2.40 to 5.85 g/100 g DW. This variation was likely due to genetic factors, soil conditions, and environmental influences. These results indicate that WMFs serve as a good source of low-fat and high-protein food.

To investigate the statistical differences in fat content among the same varieties from different regions, the fat content of THT-HN and THT-XJ was compared. The results indicated that the fat content of THT-HN (7.58 g/100 g DW) was significantly higher than that of THT-XJ (5.63 g/100 g DW) (*p* < 0.05). Furthermore, comparisons of the differences among various varieties within the same region revealed that variety had a greater influence on fat content than region. This finding aligns with the study by Pedrali et al. [9], which demonstrated that the variety exerted a greater influence on quinoa fat content than the region.

**Figure 2:**
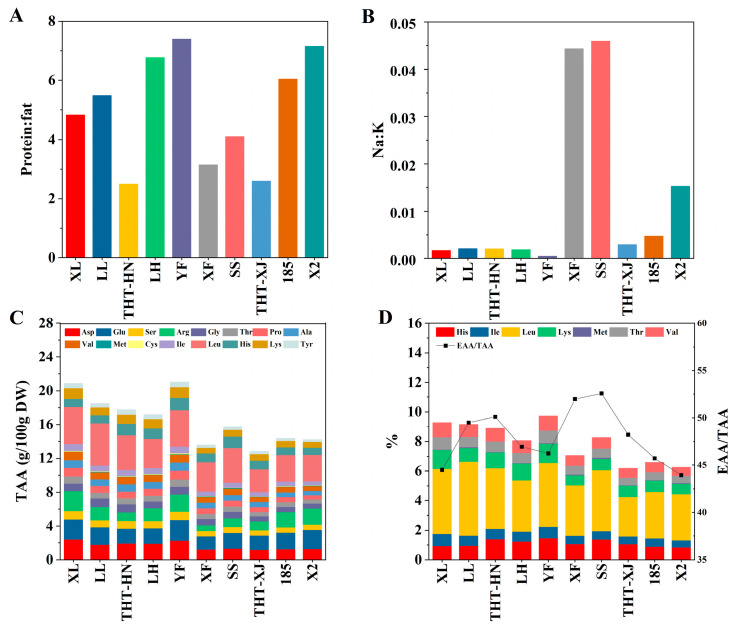
(**A**) Protein–fat ratio, (**B**) Na:K ratio, (**C**) TAA content, and (**D**) EAA content and EAA/TAA ratio of different samples of WMFs. Note: His, Histidine; Ile, Isoleucine; Leu, Leucine; Lys, Lysine; Met, Methionine; Thr, Threonine; Val, Valine; Asp, Aspartic Acid; Glu, Glutamic Acid; Ser, Serine; Arg, Arginine; Gly, Glycine; Pro, Proline; Ala, Alanine; Cys, Cysteine; Tyr, Tyrosine; EAA, essential amino acids; TAA, total amino acids.

YF exhibited the highest protein–fat ratio, while THT-HN showed the lowest (Figure 2A). These results suggest that YF holds a distinct advantage in low-fat/high-protein composition compared to other varieties, making it as a superior raw material for the development of low-fat and high-protein functional products.

### 3.3. Ash

The ash content of WMFs ranged from 8.57 to 19.09 g/100 g DW (Figure 1C). THT-HN showed the lowest ash content, while XF recorded the highest. Significant differences in ash content were observed between Henan and Xinjiang (*p* < 0.05), with Xinjiang displaying a higher range of 11.83–19.09 g/100 g DW compared to Henan’s range of 8.57–16.98 g/100 g DW.

### 3.4. Minerals

Ash content is closely associated with mineral composition. which plays an essential role in the normal functioning of organisms. Minerals play critical physiological roles in bone/soft tissue development, neuromuscular transmission, blood coagulation, and enzymatic activity [27]. Minerals are classified into macroelements (MaE) and microelements (MiE) based on daily requirements. In this study, the following elements were analyzed: MaE (calcium, magnesium, potassium, and sodium) and MiE (chromium, copper, iron, manganese, selenium, zinc, cobalt, and molybdenum). The mineral contents of ten WMF samples are summarized in Table 1. Notably, THT-XJ had the highest mineral content at 54,870.28 mg/kg DW, followed by XF at 54,596.56 mg/kg DW, whereas YF showed the lowest at 30,702.50 mg/kg DW.

#### 3.4.1. Macronutrients

Among the macroelements, potassium was the most abundant. As an essential mineral, potassium plays vital roles in maintaining normal heartbeat rhythm, regulating blood pressure, and facilitating muscle contraction. Additionally, it contributes to nerve conduction and water balance regulation. Furthermore, potassium activates specific enzymes involved in intracellular glucose metabolism and protein synthesis. The European Food Safety Authority (EFSA) recommends a daily potassium intake of 3500 mg for adults, intakes below this threshold increase stroke risk [28]. The potassium content in WMFs ranged from 27,077.91 to 45,571.46 mg/kg DW, surpassing levels found in bananas (4870–5700 mg/kg DW) and grains like quinoa (6967–12,000 mg/kg DW) [29,30,31].

Sodium plays critical roles in regulating intracellular and extracellular osmotic pressure, as well as maintaining acid-base balance. It is essential for nerve conduction, muscle contraction, and normal blood pressure maintenance. While adequate sodium intake supports various physiological functions, excessive consumption may increase the risk of hypertension, cardiovascular disease, and cerebrovascular disorders [32]. Thus, the relatively low sodium content in WMFs appears to be a beneficial characteristic. According to EFSA, a 2.0 g/day sodium intake is sufficient to maintain sodium balance in adults [33]. The sodium content in WMFs ranged from 12.51 to 1581.53 mg/kg DW. Notably, the sodium content of XF (1581.53 mg/kg DW) and SS (1408.52 mg/kg DW) was significantly higher than that of other varieties, reaching levels approximately 100-fold greater than that of THT-HN (12.51 mg/kg DW).

Furthermore, the Na:K ratios among the ten WMF samples ranged from 0.0005 to 0.0459 (Figure 2B). With the exception of XF, SS, and X2, the ratios of the other varieties ranged from 0.0005 to 0.0047, indicating nutritionally advantageous low values, as elevated Na:K ratios are associated with an increased risk of hypertension [34]. YF showed the lowest Na:K ratio, making it the optimal choice for high-potassium and low-sodium materials.

Apart from THT-HN and 185, calcium, which ranks second among macroelements, serves as the primary structural component of bones and teeth. Adequate calcium intake is crucial for bone development and also plays a vital role in regulating muscle contraction, blood coagulation, and nerve conduction. EFSA recommends a daily calcium intake of 750 mg for adults over the age of 25 [35]. The calcium content of WMFs ranged from 1076.37 to 11,734.56 mg/kg DW. Notably, XF (11,734.56 mg/kg DW) and SS (11,542.50 mg/kg DW) contained significantly higher calcium levels than the other varieties, whereas THT-HN exhibited the lowest content.

Magnesium ions serve as cofactors for numerous enzymes and interact with calcium ions to regulate bone mineral metabolism and maintain bone health. EFSA recommends a daily magnesium intake of 350 mg for adult males and 300 mg for adult females [36]. The magnesium content in WMFs ranged from 2398.53 to 4790.12 mg/kg DW, which was higher than that of Hawaiian bananas (375 mg/kg DW) [37]. XF exhibited the highest magnesium level at 4790.12 mg/kg DW, followed by SS at 4477.81 mg/kg DW. Thus, XF and SS may serve as potential sources of magnesium (and calcium) supplementation in future applications.

#### 3.4.2. Micronutrients

The micronutrient contents were highest in SS (1194.23 mg/kg DW), followed by XF (798.12 mg/kg DW), with 185 showing the lowest level (118.32 mg/kg DW). Iron, zinc, and copper were identified as the predominant micronutrients in WMFs. Iron not only constitutes myoglobin for oxygen transport but also enhances immunity and supports growth. The iron content in WMFs ranged from 39.96 to 1051.79 mg/kg DW. Zinc serves as the active center for many enzymes that play crucial roles in the metabolism of proteins, carbohydrates, and fats. Additionally, it promotes growth hormone synthesis, cell division, immune cell activation, and antibody production. The zinc content in WMFs ranged from 24.19 to 58.10 mg/kg DW.

Copper, an essential micronutrient, participates in electron transfer and acts as a core component of multiple enzymes. The copper content in WMFs varied from 10.38 to 27.23 mg/kg DW.

Manganese, another essential dietary element for mammals, acts as a cofactor in metalloenzymes involved in amino acid, lipid, and carbohydrate metabolism. The manganese content in WMFs ranged from 25.72 to 76.20 mg/kg DW.

Additionally, the cadmium content in WMFs was analyzed and found to be below 0.015 mg/kg DW.

### 3.5. Starch, Soluble Sugar, Carbohydrate, Total Dietary Fiber

A significant difference in starch content was observed between XL (30.17 mg/g DW) and THT-XJ (11.49 mg/g DW) (*p* < 0.05) (Figure 1D). The starch content of SS, THT-XJ, and X2 showed similar values, ranging from 17.74 to 18.68 mg/g DW, the other two varieties from Xinjiang exhibited starch content (22.41–22.99 mg/g DW) comparable to YF, nearly twice that of THT-HN (11.49 mg/g DW). Regarding soluble sugars, XL showed the highest content (11.04 mg/g DW), whereas X2 had the lowest (7.90 mg/g DW). Significant differences were found in the soluble sugar content of the same WMF varieties, and the soluble sugar contents of THT-HN and THT-XJ were 10.34 mg/g DW and 8.61 mg/g DW, respectively.

The soluble sugar content of WMFs ranged from 7.90 to 11.04 mg/g DW (Figure 1E), with Henan varieties ranging from 8.21 to 11.04 mg/g DW and Xinjiang varieties from 7.90 to 9.77 mg/g DW. Among these, XL exhibited the highest soluble sugar content, followed by LL (10.60 mg/g DW) and THT-HN (10.34 mg/g DW). Significant differences in soluble sugar content were observed between the same variety from Henan and Xinjiang, with THT-HN and THT-XJ containing 10.34 mg/g DW and 8.61 mg/g DW, respectively.

Carbohydrates in food provide essential energy for the human body, facilitating cellular functions. The carbohydrate content in WMFs ranged from 35.61 to 50.19 g/100 g DW (Figure 1F), indicating their value as an effective energy source. Additionally, regional comparisons revealed that THT-XJ (49.93 g/100 g DW) exceeded THT-HN (43.37 g/100 g DW). These variations may be due to environmental, geographical, and edaphic factors [9].

Dietary fiber, a non-digestible carbohydrate, plays crucial roles in enhancing gastrointestinal function and regulating blood glucose and cholesterol levels. The total dietary fiber content of WMFs ranged from 16.58 to 26.21 g/100 g DW (Figure 1G). Notably, LH exhibited significantly higher total dietary fiber content (25.15 g/100 g DW) compared to other varieties from Henan (18.76–21.61 g/100 g DW) (*p* < 0.05). Furthermore, significant differences in total dietary fiber content were observed among Xinjiang varieties (*p* < 0.05), with 185 demonstrating the highest value at 26.21 g/100 g DW, while others ranged 16.58 to 21.02 g/100 g DW.

### 3.6. Moisture

The moisture content of WMFs ranged from 6.76 to 11.89 g/100 g DW (Figure 1H). Overall, Henan varieties exhibited a moisture content between 6.76 and 7.48 g/100 g DW, while those from Xinjiang ranged from 8.89 to 11.89 g/100 g DW. WMFs from Xinjiang showed a significantly higher moisture content compared to those from Henan (*p* < 0.05). Inter-regional variations were observed within the same variety: THT-HN (6.76 g/100 g DW) had 5.6 times lower moisture content than THT-XJ (11.89 g/100 g DW). The moisture content of THT-XJ was higher than that of THT-HN. These differences may be attributed to the structural characteristics of WMFs, as well as to the climatic and light conditions in different walnut planting areas. Additionally, the plant cells of WMFs with high water content may possess slightly larger vacuoles than those with low water content, allowing them to store more water [38].

### 3.7. Amino Acids

Wang et al. [4] have shown that WMFs contain a comprehensive amino acid profile, including nine essential amino acids (EAA) for human nutrition. In this study, the amino acid content of ten WMF samples was analyzed. Total amino acid content ranged from 12.82 to 21.03 g/100 g DW, with a peak observed in YF (Figure 2C). Leucine, glutamic acid, aspartic acid, arginine, and histidine were the predominant amino acids, while the concentration of methionine and cystine was relatively low. Leucine showed the highest content, accounting for 2.67–5.01 g/100 g DW. Research has demonstrated that leucine, a branched-chain amino acid, is closely linked to type 2 diabetes, insulin resistance, obesity, and other metabolic disorders [39].

The EAA content of the ten WMF samples ranged from 6.18 to 9.72 g/100 g DW, with LH exhibiting the highest level (Figure 2D). The ratio of EAA to total amino acids (TAA) (EAA/TAA) among the WMFs ranged from 44.49% to 52.56%, and SS exhibited the highest ratio of EAA/TAA, while X2 showed the lowest. The EAA/TAA ratios of the ten WMF samples exceeded 40%. As shown in Table 2, the ratio of EAA to non-essential amino acids (NEAA) (EAA/NEAA) ranged from 78.38% to 110.81%. According to the FAO/WHO 1991 recommended model, the EAA/TAA ratio should be about 40%, and the EAA/NEAA ratio should exceed 60% for proteins to be classified as high-quality [40]. Both EAA/TAA and EAA/NEAA ratios in ten WMF samples exceeded the FAO/WHO 1991 reference pattern [41], suggesting the presence of high-quality protein. SS showed the highest ratios (EAA/TAA: 52.56%; EAA/NEAA: 110.81%), followed by XF (EAA/TAA: 51.98%; EAA/NEAA: 108.23%), indicating superior amino acid profiles compared to other samples.

To evaluate the EAA quality in WMFs, the AAS of EAA (in 1 g of the sample protein) to the reference protein (in 1 g of the standard protein) of FAO/WHO in 2013 were calculated [23,42]. As shown in Table 2, the first limiting amino acid in WMFs was methionine + cystine, while the second limiting amino acid was phenylalanine + tyrosine. The deficiency of these limiting amino acids can affect the absorption and utilization of other amino acids [43]. WMF-based products can be complemented with other food sources to compensate for inadequate levels of methionine and cystine. For example, meat and milk are rich sources of methionine [44]. According to FAO/WHO standards, AAS values approaching 1.0 indicate an optimal amino acid balance. For all EAA except methionine + cystine and phenylalanine + tyrosine, the AAS values exceeded 1.0. The essential amino acid index (EAAI) serves as a quantitative measure of protein quality by evaluating the similarity between EAA content and the reference standard. Values approaching 1.0 indicate near-ideal alignment with the reference profile. In this study, YF showed the highest EAAI value, demonstrating the closest correspondence to the reference protein pattern.

### 3.8. Polyphenols

**Figure 3:**
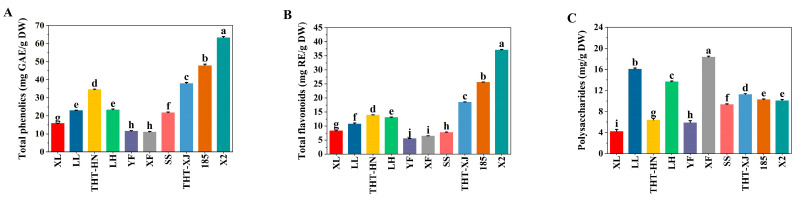
(**A**) Total phenolics, (**B**) total flavonoids, and (**C**) polysaccharides of different samples of WMFs. Note: Data are presented as means ± standard (*n* = 3), and different superscript letters are significantly different *p* < 0.05.

Beyond nutritional characteristics, the active components in WMFs were investigated. Phenolic compounds, known for their antioxidant properties, effectively scavenge free radicals and contribute significantly to antioxidant activity. The TPC in WMFs ranged from 11.12 to 63.31 mg GAE/g DW (Figure 3A). Notably, the TPC of X2 was significantly higher than that of other varieties (*p* < 0.05), with XF showing the lowest content. The highest value was 5.7-fold greater than the lowest. Additionally, TPC varied regionally within the same variety: THT-HN contained 34.60 mg GAE/g DW, whereas THT-XJ had 37.94 mg GAE/g DW. These variations may be attributed to genetic differences and climatic conditions in their respective cultivation areas [45].

### 3.9. Flavonoids

Flavonoids, as secondary metabolites, possess a variety of physiological functions, including antibacterial, antioxidant, and anti-inflammatory properties. The TFC ranged from 5.56 to 37.11 mg RE/g DW (Figure 3B), with a 6.7-fold difference between the highest and lowest values. Regionally, flavonoid levels varied within the same variety: THT-HN contained 13.91 mg RE/g DW, whereas THT-XJ showed 18.50 mg RE/g DW. Overall, WMFs from Xinjiang exhibited higher TPC and TFC than those from Henan, and X2 exhibited the highest TPC and TFC. This variation may be attributed to factors such as climate, soil conditions, light exposure, and genetic factors [46].

According to Muzaffer and Paul [5], the TPC in WMFs ranged from 122.1 to 129.76 mg GAE/g DW, while the TFC ranged from 131.79 to 144.62 mg RE/g DW. Żurek et al. [47] reported comparable levels of 248.33 ± 2.33 mg GAE/g DW for polyphenols and 111.01 ± 1.03 mg RE/g DW for flavonoids. Both studies demonstrated significantly higher values than those obtained in our current research. In contrast, Wang et al. [4] measured substantially lower concentrations, with the TPC ranging from 21.2 to 24.3 mg GAE/g DW and the TFC from 14.4 to 21.5 mg RE/g DW. These discrepancies may stem from variations in WMF genotypes, cultivation conditions (including climate and soil composition), and differences in extraction methodologies [48].

### 3.10. Polysaccharides

Polysaccharides exhibit various bioactive properties, including antioxidant, antitumor, and hypoglycemic effects. The polysaccharide content ranged from 4.41 to 18.35 mg glucose/g DW (Figure 3C), with XF showing the highest level at 18.35 mg glucose/g DW and THT-HN showing the lowest at 4.41 mg glucose/g DW, representing a 4.2-fold difference. Regional variations in polysaccharide content were observed within the same variety. THT-XJ (11.23 mg glucose/g DW) exhibited a 2.5-fold higher level than THT-HN (4.41 mg glucose/g DW). Except for THT-HN, the polysaccharide content in WMFs surpassed that of Polish rose petals [49].

### 3.11. In Vitro Antioxidant Capacity

**Figure 4:**
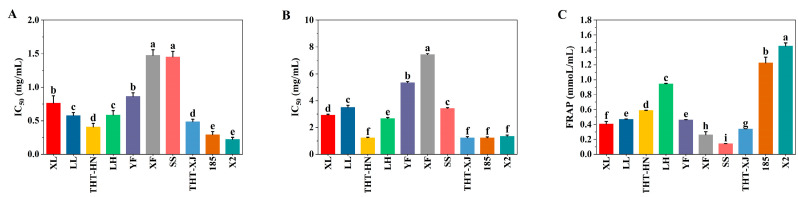
Antioxidant capacity of different samples of WMFs: (**A**) DPPH radical scavenging capacity, (**B**) ABTS radical scavenging capacity, and (**C**) FRAP. Note: Different superscript letters are significantly different *p* < 0.05. DPPH, 2,2-diphenyl-1-picrylhydrazyl; ABTS, 2,2′-Azinobis-(3-ethyl-benzothiazoline-6-sulfonic acid); FRAP, ferric reducing antioxidant power.

To evaluate the antioxidant properties of WMF ethanolic extracts, DPPH and ABTS radical scavenging capacity and FRAP were assessed. The IC_50_ values, which are inversely proportional to antioxidant activity capacity, were determined for DPPH and ABTS radical scavenging capacity (Figure 4A,B). Among the WMF varieties, X2 demonstrated the strongest DPPH radical scavenging capacity (IC_50_ = 0.22 mg/mL), while XF exhibited the weakest capacity (IC_50_ = 1.47 mg/mL). The DPPH radical scavenging activities of THT-HN (IC_50_ = 0.41 mg/mL) and THT-XJ (IC_50_ = 0.49 mg/mL) were similar. Notably, the DPPH radical scavenging capacity of WMF polyphenol extracts surpassed that of the Algerian *Ficus carica* L. (IC_50_ = 3.11 mg/mL) [50].

For ABTS radical scavenging capacity, 185, THT-HN, and THT-XJ showed the lowest IC_50_ values (1.23 mg/mL), closely followed by X2 (1.33 mg/mL), indicating the strongest ABTS radical scavenging capacity. In contrast, XF exhibited significantly higher IC_50_ values (7.43 mg/mL). The results demonstrated that no statistically significant differences were observed in the IC_50_ values of DPPH and ABTS radical scavenging capacity among the same variety of WMFs.

Given that a substance’s antioxidant capacity is directly correlated with its reducing power, the FRAP assay can be used to assess its antioxidant capacity, with results from the FRAP assay being directly proportional to antioxidant capacity [51]. In this study, X2 demonstrated the strongest iron-reducing power (1.45 mmol/L), whereas SS showed the weakest (0.14 mmol/L), followed by XF (0.26 mmol/L) (Figure 4C). Overall, X2 exhibited the strongest antioxidant capacity, while XF displayed relatively weaker capacity. However, it is important to note that these antioxidant assays (DPPH, ABTS, FRAP) serve as indicators of potential bioactivity but do not directly predict effects in enzymatic or cellular systems.

### 3.12. Correlation Analysis Between Antioxidant Capacity and Bioactive Compounds

**Figure 5:**
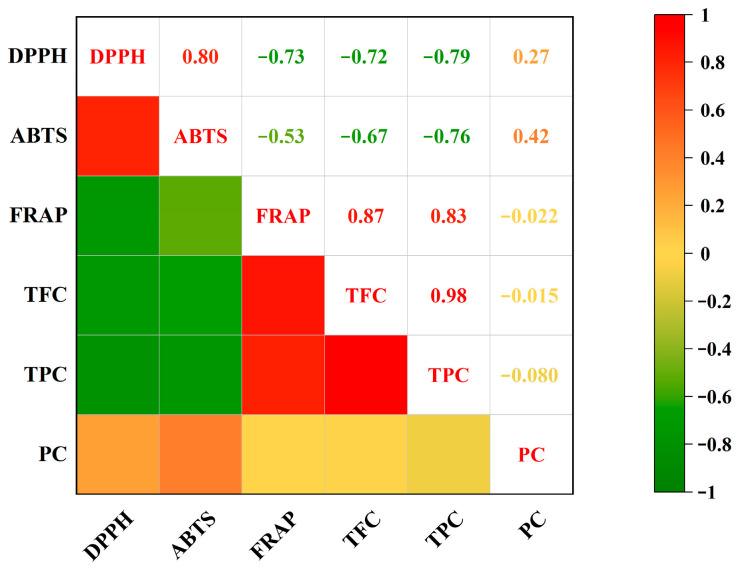
Correlation analysis between TPC, TFC, PC, and antioxidant capacity. Note: TPC, total phenolic content; TFC, total flavonoid content; PC, polysaccharide content; DPPH, 2,2-diphenyl-1-picrylhydrazyl; ABTS, 2,2′-Azinobis-(3-ethyl-benzothiazoline-6-sulfonic acid); FRAP, ferric reducing antioxidant power.

TPC and TFC have a substantial correlation with antioxidant capacity [52]. In this study, the Pearson correlation coefficients between bioactive components, including polyphenols, flavonoids, and polysaccharides, and antioxidant activities of ten WMF samples were calculated. The IC_50_ value of DPPH radical scavenging capacity showed a highly statistically significant negative correlation with TPC (*r* = −0.79, *p* < 0.01) (Figure 5), while a highly statistically significant positive correlation was observed with the IC_50_ value of ABTS radical scavenging capacity (*r* = 0.80, *p* < 0.01). Furthermore, TFC showed a statistically significant negative correlation with FRAP values (*r* = −0.72, *p* < 0.05).The IC_50_ value of ABTS radical scavenging capacity showed statistically significant negative correlation with the TPC (*r* = −0.76, *p* < 0.05) and TFC (*r* = −0.67, *p* < 0.05). Conversely, FRAP values exhibited highly statistically significant positive correlation with TPC (*r* = 0.83, *p* < 0.01) and TFC (*r* = 0.87, *p* < 0.01). Collectively, both TPC and TFC were strongly associated with antioxidant capacity, whereas polysaccharide content showed no significant correlation with antioxidant capacity. However, their specific bioactivities and underlying mechanisms require further validation.

### 3.13. Multivariate Statistical Analysis

**Figure 6:**
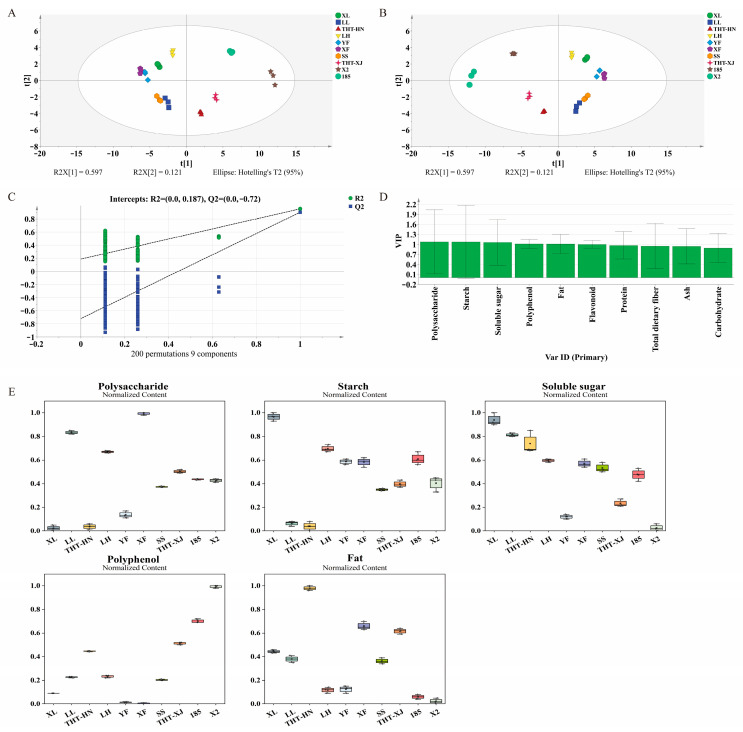
Multivariate statistical analysis of different samples of WMFs: (**A**) PCA score plot; (**B**) PLS-DA score plot; (**C**) cross-validation plot of the PLS-DA model with 200 permutations, (**D**) VIP score plot; and (**E**) boxplots of the normalized relative content of different nutritional components. Note: PCA, principal component analysis; PLS-DA, partial least squares-discriminant analysis; VIP, variable importance in projection. Normalized intensities are presented in *y*-axis in Figure 6E.

PCA is an unsupervised multivariate statistical analysis. In this study, PCA was performed using the nutritional components of ten WMF samples. The PCA model revealed that principal component 1 (PC_1_) accounted for 59.7% of the variance, while principal component 2 (PC_2_) contributed 12.1%, indicating effective model representation of the original data (Figure 6A). 185 and X2 (both from Xinjiang) were located in the upper right quadrant. In the upper left quadrant were LH, XL, and YF (all from Henan), along with XF (from Xinjiang). SS (from Henan) and LL (from Henan) were found in the lower left quadrant. Notably, the THT varieties from different regions (THT-HN and THT-XJ) were clustered together within the same quadrant. This indicates that their nutritional profiles were more similar to each other than to other varieties from the same region, highlighting the predominant role of genetic variety over geographical origin.

Furthermore, PLS-DA, which is a supervised multivariate statistical analysis, was employed to evaluate variables with significant contributions to classification (Figure 6B). The PLS-DA model parameters (R^2^X = 1, R^2^Y = 0.937, Q^2^ = 0.886) indicated excellent fitness and good predictability [53]. A permutation test at 200 times was conducted, and the intercept values of R^2^ and Q^2^ were 0.187 and −0.72, respectively, demonstrating no over-fitting of the PLS-DA model (Figure 6C). A variable importance in projection (VIP) score greater than 1 and a *p*-value less than 0.05 were used to define potential markers for distinguishing sample differences [54]. The *p*-values were derived from the omics function in SIMCA software, and the results are as shown in Table S1. Consequently, polysaccharide, starch, soluble sugar, polyphenol, and fat content with VIP > 1 and *p* < 0.05 were identified as potential markers for WMFs varietal discrimination (Figure 6D).

The boxplots in Figure 6E showed how these markers (polysaccharides, starch, soluble sugars, polyphenols, and fats) were distributed throughout the samples. The results showed that the five markers exhibited significant differences across the ten WMF samples. Among them, the box for polysaccharides was the highest, indicating the greatest dispersion among the samples. The content of the XF variety (18.35 mg glucose/g DW) was 4.2 times higher than that of the THT-HN variety (4.41 mg glucose/g DW), and significant differences were also observed between different regions for the same variety. The distribution of the starch boxplot was concentrated, with a relatively low median, showing a characteristic of “a few high values (e.g., the XL variety at 30.17 mg/g DW) and mostly low values (e.g., the THT-XJ variety at 11.49 mg/g DW).” In contrast, soluble sugars exhibited the narrowest interquartile range and the most stable content (7.90–11.04 mg/g DW), with a less than 1.4-fold difference between the highest and lowest values. This stability makes them a potential marker for differentiating varieties, effectively complementing other highly volatile components. Although the content distributions of polyphenols and fat are not fully presented in the figure, these compounds, together with the components mentioned above, collectively form a characteristic nutritional profile for identifying WMF varieties.

## 4. Conclusions

This study provides the first comprehensive simultaneous comparative analysis of ten walnut male flower varieties across two major geographical regions (Henan and Xinjiang), establishing a robust database for understanding genotype–environment interactions in WMFs. The results revealed that genetic factors (variety) exerted greater influence on nutritional composition than regional environmental conditions. As a foundational screening study, our integrated analysis of nutritional components, mineral profiles, and in vitro antioxidant capacity revealed common nutritional characteristics across all WMF samples. Specifically, they were rich in protein, minerals (notably potassium and calcium), and amino acids (particularly leucine), while exhibiting low fat content and a favorable Na/K ratio. The high levels of polyphenols, flavonoids, along with strong antioxidant capacity, measured by spectrophotometric assays, collectively indicate the potential of WMFs as functional ingredients. However, their specific bioactivities and underlying mechanisms require further validation through more targeted studies.

It is noteworthy that LL exhibited the highest protein content, whereas X2 showed the highest levels of polyphenols and flavonoids and the strongest antioxidant capacity. The PLS-DA model revealed that polysaccharide, starch, soluble sugar, polyphenol, and fat are key markers for differentiating WMF varieties. Building upon the mineral and nutritional baseline established in this study, future research should employ advanced techniques such as liquid chromatography–tandem mass spectrometry (LC-MS) and gas chromatography–mass spectrometry (GC-MS) to precisely identify specific organic metabolites (e.g., phenolic compounds and lipids) and further validate the health benefits. All samples were collected during the same growing season. While this approach aids in isolating the effects of variety and region by controlling for interannual climatic variation, future studies incorporating samples from multiple years would be valuable for assessing the stability of these nutritional and bioactive constituents under varying environmental factors. This study provides essential nutritional references for the application of WMFs and supports their potential as functional food ingredients with health benefits.

## Figures and Tables

**Table 1:** Mineral content of different samples of WMFs.

Element	XL	LL	THT-HN	LH	YF	XF	SS	THT-XJ	185	X2
	**mg/kg DW**									
Macroelements (MaE)										
Na	59.31 ± 2.26 ^g^	91.58 ± 1.25 ^f^	12.51 ± 1.48 ^h^	68.55 ± 1.75 ^fg^	66.27 ± 4.03 ^fg^	1581.53 ± 50.46 ^a^	1408.52 ± 19.48 ^b^	134.66 ± 5.20 ^e^	188.18 ± 3.25 ^d^	468.27 ± 4.94 ^c^
Mg	2547.55 ± 25.87 ^h^	2907.10 ± 63.80 ^g^	2398.53 ± 15.57 ^e^	3026.88 ± 44.27 ^f^	3002.65 ± 51.76 ^fg^	4790.12 ± 158.46 ^a^	4477.81 ± 48.98 ^b^	3636.49 ± 47.58 ^d^	3816.66 ± 51.49 ^c^	3506.52 ± 48.50 ^e^
K	35,925.42 ± 1490.42 ^e^	43,045.06 ± 893.92 ^b^	27,077.91 ± 410.33 ^d^	33,080.97 ± 539.28 ^f^	34,869.16 ± 1478.69 ^e^	35,692.22 ± 1078.31 ^e^	30,673.59 ± 64.16 ^g^	45,571.46 ± 149.60 ^a^	39,896.38 ± 380.18 ^c^	30,720.37 ± 794.43 ^g^
Ca	4626.22 ± 72.94 ^d^	5258.52 ± 91.90 ^c^	1076.37 ± 75.91 ^g^	4100.54 ± 211.94 ^e^	5420.38 ± 146.93 ^c^	11734.56 ± 405.95 ^a^	11542.50 ± 71.81 ^a^	5393.63 ± 244.64 ^c^	3240.19 ± 173.50 ^f^	8660.77 ± 551.57 ^b^
∑ MaE	43,158.50	51,302.26	40,276.93	43,358.47	30,565.32	53,798.43	48,102.42	54,736.25	47,141.40	43,355.92
Microelements (MiE)										
Cr	0.50 ± 0.02 ^e^	0.77 ± 0.02 ^d^	1.09 ± 0.08 ^c^	0.58 ± 0.01 ^de^	0.61 ± 0.05 ^de^	7.59 ± 0.31 ^b^	8.24 ± 0.15 ^a^	n.d.	n.d.	n.d.
Mn	57.75 ± 0.62 ^cd^	47.30 ± 2.41 ^e^	32.80 ± 1.12 ^d^	45.93 ± 1.52 ^e^	64.05 ± 0.60 ^b^	59.72 ± 0.55 ^c^	76.20 ± 2.17 ^a^	25.72 ± 1.07 ^g^	27.13 ± 0.50 ^g^	39.01 ± 0.17 ^f^
Fe	94.51 ± 6.70 ^f^	151.49 ± 0.40 ^de^	39.96 ± 1.64 ^d^	140.89 ± 10.18 ^e^	137.37 ± 5.34 ^e^	669.68 ± 4.52 ^b^	1051.79 ± 20.39 ^a^	67.11 ± 1.07 ^g^	54.85 ± 3.21 ^g^	278.43 ± 9.04 ^c^
Cu	20.86 ± 0.17 ^c^	27.23 ± 0.11 ^a^	22.05 ± 0.93 ^a^	19.54 ± 0.02 ^d^	22.66 ± 0.44 ^b^	18.22 ± 0.15 ^e^	16.99 ± 0.25 ^f^	16.68 ± 0.56 ^f^	12.66 ± 0.17 ^g^	10.38 ± 0.02 ^h^
Zn	51.05 ± 0.66 ^c^	58.10 ± 1.12 ^b^	41.24 ± 1.53 ^a^	51.78 ± 1.16 ^c^	57.97 ± 0.52 ^b^	42.07 ± 0.37 ^d^	40.52 ± 0.67 ^d^	24.19 ± 1.06 ^f^	23.43 ± 0.28 ^f^	26.28 ± 0.20 ^e^
Se	0.06 ± 0.01 ^d^	0.10 ± 0.02 ^d^	0.02 ± 0.00 ^d^	0.03 ± 0.02 ^d^	0.09 ± 0.01 ^d^	0.82 ± 0.02 ^a^	0.49 ± 0.02 ^b^	0.31 ± 0.14 ^c^	0.25 ± 0.04 ^c^	0.48 ± 0.04 ^b^
Mo	0.01 ± 0.00 ^e^	0.36 ± 0.00 ^c^	0.01 ± 0.00 ^b^	0.68 ± 0.00 ^a^	0.32 ± 0.01 ^d^	0.01 ± 0.00 ^e^	0.01 ± 0.00 ^e^	0.01 ± 0.00 ^e^	0.01 ± 0.00 ^e^	0.01 ± 0.00 ^e^
∑ MiE	224.74	285.35	259.44	283.06	137.17	798.12	1194.23	134.02	118.34	354.59
Cd	0.006 ± 0.00 ^c^	0.011 ± 0.00 ^b^	n.d.	0.010 ± 0.00 ^b^	0.015 ± 0.00 ^a^	0.002 ± 0.00 ^d^	n.d.	0.004 ± 0.00 ^d^	0.003 ± 0.00 ^d^	0.006 ± 0.00 ^c^
Total content	43,383.25	51,587.61	40,536.37	43,641.54	30,702.50	54,596.56	49,296.65	54,870.28	47,259.75	43,710.52

Note: XL, Xiangling; LL, Lvling; THT-HN, Tuhetao from Henan; YF, Yufeng; LH, Liaohe; XF, Xingfu; SS, Sansan; THT-XJ, Tuhetao from Xinjiang; X2, Xin2. Na, Sodium; Mg, Magnesium; K, Potassium; Ca, Calcium; Cr, Chromium; Mn, Manganese; Fe, Iron; Cu, Copper; Zn, Zinc; Se, Selenium; Mo, Molybdenum; Cd, Cadmium. n.d., not detected. Data are shown as mean ± standard deviation values of triplicate measurements. Different letters (in the same row) denote a statistically significant difference at *p* < 0.05.

**Table 2:** EAA composition of compared to the FAO/WHO 2013 pattern (mg/g protein DW), EAA/NEAA, and EAAI of different samples of WMFs.

	ASS								EAA/NEAA (%)	EAAI
	His	Ile	Leu	Lys	Met + Cys	Phe + Tyr	Thr	Val		
XL	2.78 ± 0.01 ^i^	1.29 ± 0.02 ^bc^	3.44 ± 0.03 ^c^	1.29 ± 0.01 ^b^	0.20 ± 0.00 ^d^	0.61 ± 0.01 ^d^	1.61 ± 0.00 ^b^	1.17 ± 0.02 ^c^	80.16 ± 0.31 ^f^	0.78 ± 0.01 ^e^
LL	2.68 ± 0.00 ^h^	1.02 ± 0.03 ^e^	3.72 ± 0.06 ^b^	0.88 ± 0.01 ^f^	0.24 ± 0.01 ^bc^	0.50 ± 0.00 ^bc^	1.28 ± 0.01 ^f^	0.94 ± 0.03 ^b^	98.03 ± 1.05 ^b^	0.67 ± 0.00 ^g^
THT-HN	4.90 ± 0.01 ^c^	1.37 ± 0.03 ^a^	3.81 ± 0.15 ^b^	1.28 ± 0.01 ^b^	0.23 ± 0.00 ^c^	0.77 ± 0.01 ^c^	1.63 ± 0.04 ^b^	1.26 ± 0.03 ^b^	100.46 ± 3.76 ^b^	0.84 ± 0.01 ^b^
LH	4.22 ± 0.01 ^e^	1.26 ± 0.02 ^c^	3.10 ± 0.12 ^d^	1.28 ± 0.01 ^b^	0.26 ± 0.01 ^b^	0.67 ± 0.01 ^b^	1.54 ± 0.01 ^d^	1.13 ± 0.02 ^d^	88.49 ± 2.52 ^d^	0.79 ± 0.01 ^d^
YF	4.51 ± 0.01 ^d^	1.33 ± 0.04 ^ab^	3.52 ± 0.04 ^c^	1.36 ± 0.01 ^a^	0.28 ± 0.01 ^a^	0.70 ± 0.01 ^a^	1.72 ± 0.02 ^a^	1.22 ± 0.04 ^c^	86.04 ± 0.69 ^de^	0.86 ± 0.01 ^a^
XF	3.98 ± 0.00 ^f^	1.11 ± 0.04 ^d^	3.37 ± 0.03 ^c^	0.84 ± 0.01 ^g^	0.24 ± 0.01 ^bc^	0.46 ± 0.00 ^bc^	1.41 ± 0.02 ^e^	1.05 ± 0.04 ^c^	108.23 ± 1.15 ^a^	0.68 ± 0.00 ^g^
SS	5.37 ± 0.01 ^a^	1.25 ± 0.04 ^c^	4.25 ± 0.16 ^a^	1.03 ± 0.01 ^d^	0.30 ± 0.02 ^a^	0.55 ± 0.01 ^a^	1.57 ± 0.02 ^cd^	1.17 ± 0.04 ^a^	110.81 ± 2.51 ^a^	0.80 ± 0.01 ^c^
THT-XJ	5.12 ± 0.00 ^b^	1.36 ± 0.02 ^a^	3.40 ± 0.06 ^c^	0.27 ± 0.01 ^b^	0.16 ± 0.01 ^e^	0.55 ± 0.00 ^e^	1.57 ± 0.01 ^c^	1.23 ± 0.02 ^c^	93.12 ± 1.10 ^c^	0.74 ± 0.00 ^f^
185	3.99 ± 0.01 ^f^	1.33 ± 0.02 ^ab^	3.73 ± 0.13 ^b^	1.14 ± 0.01 ^c^	0.23 ± 0.01 ^c^	0.53 ± 0.01 ^c^	1.57 ± 0.01 ^cd^	1.16 ± 0.02 ^b^	84.27 ± 1.79 ^e^	0.77 ± 0.01 ^e^
X2	3.44 ± 0.00 ^g^	1.03 ± 0.01 ^e^	3.37 ± 0.13 ^c^	0.94 ± 0.01 ^e^	0.26 ± 0.01 ^b^	0.41 ± 0.00 ^b^	1.31 ± 0.01 ^f^	0.92 ± 0.01 ^c^	78.38 ± 1.88 ^f^	0.66 ± 0.00 ^h^

Note: AAS, amino acid scores; EAA, essential amino acids; NEAA, non-essential amino acids; EAAI, essential amino acid index; His, Histidine; Ile, Isoleucine; Leu, Leucine; Lys, Lysine; Met, Methionine; Cys, Cysteine; Phe, Phenylalanin; Tyr, Tyrosine; Thr, Threonine; Val, Valine. HN, Henan; XJ, Xinjiang; XL, Xiangling; LL, Lvling; THT-HN, Tuhetao from Henan; YF, Yufeng; LH, Liaohe; XF, Xingfu; SS, Sansan; THT-XJ, Tuhetao from Xinjiang; X2, Xin2. Different letters (in the same column) denote a statistically significant difference at *p* < 0.05.

## Data Availability

The original contributions presented in this study are included in the article/Supplementary Materials. Further inquiries can be directed to the corresponding authors.

## Supplementary Materials

The following supporting information can be downloaded at: https://www.mdpi.com/article/10.3390/foods14244250/s1, Table S1: *p*-values for the various nutritional components of the WMF samples.
