# Supplementary material for: Comprehensive Analysis in the Nutritional Composition, Bioactive Contents, and Antioxidant Capacity of Walnut (Juglans regia L.) Male Flowers
Source: Foods. 2025 Dec 10;14(24):4250. doi: 10.3390/foods14244250 (PMC12732885; doi:10.3390/foods14244250)
Supplement: Supplementary file 1 [file foods-14-04250-s001.zip › foods-4035979-supplementary.pdf]

Table S1. *p*-values for the various nutritional components of the WMF samples

Var ID	Probability (LL)	Probability (THT-HN)	Probability (LH)	Probability (YF)	Probability (XF)	Probability (SS)	Probability (THT-XJ)	Probability (185)	Probability (X2)
Protein	0.0113	0.0003	0.0005	0.0593	0.0003	0.0001	0.0000	0.0000	0.0000
Fat	0.0388	0.0000	0.0000	0.0001	0.0005	0.0104	0.0005	0.0000	0.0000
Ash	0.0000	0.0000	0.0016	0.0000	0.0000	0.0000	0.0000	0.0095	0.0013
Total dietary fiber	0.7108	0.0305	0.0005	0.3378	0.0227	0.0464	0.0181	0.0011	0.6544
Carbohydrate	0.0151	0.3165	0.0042	0.0274	0.0004	0.1902	0.0001	0.2272	0.0007
Starch	0.0000	0.0000	0.0006	0.0001	0.0002	0.0000	0.0000	0.0006	0.0002
Soluble sugar	0.0150	0.0296	0.0004	0.0000	0.0005	0.0004	0.0000	0.0004	0.0000
Polysaccharide	0.0000	0.0017	0.0000	0.0088	0.0000	0.0000	0.0000	0.0000	0.0000
Flavonoid	0.0007	0.0000	0.0000	0.0001	0.0003	0.0378	0.0000	0.0000	0.0000
Polyphenol	0.0000	0.0000	0.0000	0.0000	0.0000	0.0009	0.0000	0.0000	0.0000
